# Parametric Response Maps of Perfusion MRI May Identify Recurrent Glioblastomas Responsive to Bevacizumab and Irinotecan

**DOI:** 10.1371/journal.pone.0090535

**Published:** 2014-03-27

**Authors:** Domenico Aquino, Anna Luisa Di Stefano, Alessandro Scotti, Lucia Cuppini, Elena Anghileri, Gaetano Finocchiaro, Maria Grazia Bruzzone, Marica Eoli

**Affiliations:** 1 Neuro-Radiology Unit, Fondazione IRCCS Istituto Neurologico C. Besta, Milan, Italy; 2 General Neurology Unit, Fondazione IRCCS Istituto Neurologico Nazionale C. Mondino, Pavia, Italy; 3 Molecular Neuro-Oncology Unit, Fondazione IRCCS Istituto Neurologico C. Besta, Milan, Italy; University of California-San Francisco, United States of America

## Abstract

**Background:**

Perfusion weighted imaging (PWI) can be used to measure key aspects of tumor vascularity in vivo and recent studies suggest that perfusion imaging may be useful in the early assessment of response to angiogenesis inhibitors. Aim of this work is to compare Parametric Response Maps (PRMs) with the Region Of Interest (ROI) approach in the analysis of tumor changes induced by bevacizumab and irinotecan in recurrent glioblastomas (rGBM), and to evaluate if changes in tumor blood volume measured by perfusion MRI may predict clinical outcome.

**Methods:**

42 rGBM patients with KPS ≥50 were treated until progression, as defined by MRI with RANO criteria. Relative cerebral blood volume (rCBV) variation after 8 weeks of treatment was calculated through semi-automatic ROI placement in the same anatomic region as in baseline. Alternatively, rCBV variations with respect to baseline were calculated into the evolving tumor region using a voxel-by-voxel difference. PRMs were created showing where rCBV significantly increased, decreased or remained unchanged.

**Results:**

An increased blood volume in PRM (PRM_CBV+)_ higher than 18% (first quartile) after 8 weeks of treatment was associated with increased progression free survival (PFS; 24 versus 13 weeks, *p* = 0.045) and overall survival (OS; 38 versus 25 weeks, *p* = 0.016). After 8 weeks of treatment ROI analysis showed that mean rCBV remained elevated in non responsive patients (4.8±0.9 versus 5.1±1.2, p = 0.38), whereas decreased in responsive patients (4.2±1.3 versus 3.8±1.6 p = 0.04), and re-increased progressively when patients approached tumor progression.

**Conclusions:**

Our data suggest that PRMs can provide an early marker of response to antiangiogenic treatment and warrant further confirmation in a larger cohort of GBM patients.

## Introduction

Glioblastomas (GBM) are highly vascularized tumors, leading to development of therapeutic strategies targeting tumor angiogenesis [1]. Bevacizumab, a monoclonal antibody targeting the vascular endothelial growth factor (VEGF), has recently entered into the clinical arena and represents the front-runner among currently available antiangiogenic drugs [2]. Despite the significant number of studies based on GBM treatment with bevacizumab, alone or in combination with other drugs, in vivo modifications induced by treatment are poorly defined [3]. Moreover, although the highly variable response to bevacizumab, currently there are no prospectively validated predictive or prognostic biomarkers for it [4].

Perfusion weighted imaging (PWI) can be used to measure key aspects of tumor vascularity in vivo and recent studies suggest that perfusion imaging may be useful in the early assessment of response to angiogenesis inhibitors. Sorensen, studying recurrent GBM patients treated with cediranib, an inhibitor of the VEGF receptor tyrosine kinases, calculated a “vascular normalization index” by combining Ktrans (the rate of transfer of the contrast agent (CA)), microvessel volume and circulating collagen IV and found that this index (measured 1 day after treatment initiation) was predictive of overall and progression-free survival (OS and PFS) [5].

Cha et al. studied 18 patients with recurrent malignant gliomas treated with both thalidomide (an antiangiogenic agent) and carboplatin: changes in relative Cerebral Blood Volume (rCBV) are better correlated with treatment response than enhancing tumor size [6].

In 16 patients with recurrent GBM treated with bevacizumab, Sawlani observed that mean rCBV, mean leakage coefficient and hyperperfusion volume (HPV), defined as the fraction of tumor with an rCBV above a pre-specified threshold, correlate with time to progression [7].

Parametric Response Maps (PRM) are voxel-wise analytic approach to quantify significant regional changes in tumor physiology after therapy [8], [9].

Aim of this work is to compare PRMs with the classical Region Of Interest (ROI) approach [10] in the analysis of tumor changes induced by bevacizumab and irinotecan in recurrent GBM, and to evaluate if changes in tumor blood volume measured by perfusion MRI may predict clinical outcome [11].

## Methods

### Ethics statement

All patients in the current work were part of a study carried out according to the Italian Decree Law of May 8^th^, 2003 allowing treatment of patients with no other therapeutic option, with drugs not yet approved by the Italian Regulatory Agency, but with evidence of efficacy in phase II clinical trials [11]. The protocol was approved by the Ethics Committee of the Neurological Institute “Carlo Besta” of Milan and registered in the Institute database (#1/08). All patients gave written informed consent. All clinical investigation were conducted according to the principles expressed in the Declaration of Helsinki.

### Patients

Forty-two of these patients who underwent the same MRI protocol, were enrolled [11]. All patients underwent prior surgery and radiochemotherapy according to the Stupp's protocol [12], followed by second or third line chemotherapy.

Magnetic Resonance Imaging (MRI) was performed with a 1.5-T MR Unit (Magnetom Avanto; Siemens, Erlangen, Germany) before starting therapy and followed up every 8 weeks until tumor progression or treatment discontinuation.

RANO criteria were used to assess tumor response and tumor progression; however, to assess changes of FLAIR hyperintensity, a threshold of 25% or more of the maximal cross-sectional area was used [13], [14]. Baseline tumor volumes were determined on 3D post-gadolinium T1 weighted images by manually outlining the enhancing portion of the lesion using MRIcro (http://www.mccauslandcenter.sc.edu/mricro/). The total enhancing volume was obtained as the product of the number of enhancing voxels and the voxel volume.

We used the following four criteria to assess MR patterns of disease at baseline and at progression [15]. Local disease: unifocal, contiguous with the primary site or resection cavity or within a 3 cm margin. Distant disease: a second non-contiguous lesion in addiction to disease at the primary site. Multifocal disease: three or more non-contiguous lesions including the primary site (cerebrospinal fluid spread of disease was also defined as multifocal disease). Diffuse disease: extending more than 3 cm beyond the primary site with poor or indistinct contrast enhancement or FLAIR margins.

### Acquisition protocol

The radiological MR protocol included: 1) a 3D post-contrast MPRAGE T1-weighted sequence (TR\TE\TI = 1160\4.21\600 ms, matrix = 384×512, voxel size = 0.47×0.47×0.9 mm, 192 slices, AT = 1.42 min); 2) a 3D FLAIR sequence (TR\TE = 5000\477 ms, matrix = 256×256, voxel size = 1×1×1, 160 slices); 3) PWI was performed with a Dynamic Susceptibility Contrast (DSC) GRE echo-planar (EPI) sequence (TR\TE = 2040\53 ms, flip angle = 30°, matrix size = 128×128, voxel size = 2×2×5 mm, 50 dynamic volumes of 17, AT = 1.42 min). Acquisitions were carried out during the injection of the gadolinium-based contrast agent Gadovist (Bayer), 1 mol/L. A bolus injection of 9 cc was administered at 5 mL/s using an automated injector. To minimize T1-shortening effects, a contrast agent predose of 3 cc was used to saturate leaky tissue from the blood–brain barrier breakdown.

### Post-processing

Every DSC-MRI volume was spatially co-registered to the first one at baseline by an affine 12 DOF registration. Post-contrast T1-weighted images were co-registered and resampled to match the spatial resolution of the DSC volume of reference.

DSC-MRI data were processed to create rCBV maps. NordicICE (http://www.nordicneurolab.com) was used for perfusion processing, including a CA leakage correction caused by blood-brain barrier disruption, and an automated gamma-variate fitting of first-pass CA concentration curves.

The following two methods were used to evaluate rCBV variation during treatment.

#### Region Of Interest (ROI)

Three separated ROIs were placed in regions of highest perfusion seen on the rCBV color maps at baseline. Size and morphology of ROIs were maintained constant (circular 40 mm^2^ area) and the maximum value recorded. Reference ROI was drawn on contralateral normal white matter, with the same size and position [10], [16]. rCBV variation after 8 weeks of treatment was calculated through semi-automatic ROI placement in the same anatomic region as baseline.

#### Parametric Response Maps (PRMs)

This technique was previously used to assess DWI variation at two end-points for each patient after radiotherapy in GBM [17] and create rCBV maps of patients with grade III and IV gliomas receiving concurrent radiochemotherapy [8], [9].

Two ROIs were drawn by an experienced neuroradiologist on contrast-enhanced T1-weighted images: the first including all tissues into the enhancement area, the second covering the hyperintense area that was judged to be necrotic. This second volume was then subtracted from the first one and the selected region defined as the “tumor region”. A control ROI of the same size was drawn contralaterally on frontal, normal white matter.

**Figure 1:**
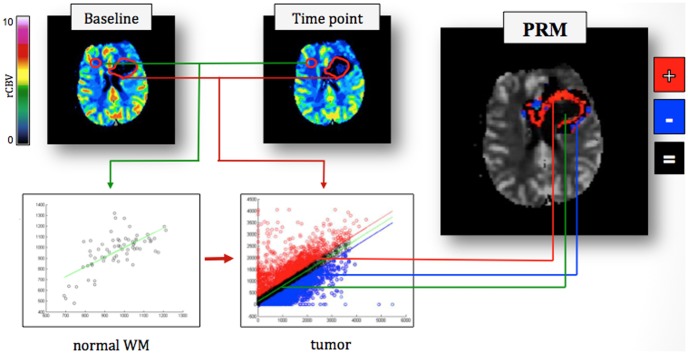
Parametric Response Maps creation process. 1) comparison of the rCBV values of a ROI placed on the normal white matter between the baseline and the time point under examination; 2) Classification of increased, decreased and unchanged differences values in the tumor area on the basis of the previously determined thresholds; 3) Chromatic representation of the difference map obtained by the subtraction of the baseline map from the time point one. Red voxels indicate an increase of rCBV, blue a decrease and black voxels are unchanged.

Perfusion changes over time were quantified by using a voxel-by-voxel analysis in the tumor region, drawn at baseline [17]. In case of progression another ROI was drawn on the new tumor volume and the sum of this ROI and baseline ROI was considered as definitive ROI. The rCBV values of each voxel within the tumor at week 8 and at time of progression were compared with baseline values. To evaluate their difference two thresholds were set: they were determined to be the 95% confidence intervals (C.I.) (1.96xSD) obtained comparing the rCBV values of the two time points in the normal contralateral white matter. The tumor region was then subdivided in three regions represented with different colors: 1) areas with rCBV greater than the upper threshold (increased CBV, iCBV), represented in red; 2) areas with rCBV lower than the lower threshold (decreased CBV, dCBV), represented in blue; 3) areas unchanged (uCBV), represented in black (Figure 1). Colored maps were than overlayed/merged on T2 reference images, allowing a qualitative assessment of perfusion changes in pathological areas. The procedure was repeated at each time point until progression.

### Statistical analysis

PFS and OS were calculated from treatment onset until disease progression or death/last follow-up, if censored. Kaplan Meier analysis estimated PFS and OS. The log rank test assessed differences in progression or survival in patients with different clinical or radiological parameters. These parameters were set at the 25°, 50°, 75°, 90° percentile and separately evaluated in all patients.

Correlations between radiological and clinical parameters or treatment response were assessed using the Mann-Whitney exact U test. The Wilcoxon rank sum test evaluated differences among radiological parameters at baseline, week 8 or progression. All p-values were two-sided.

A multivariate analysis and a Cox proportional hazard regression model analysis were performed on variables showing statistically significant differences at univariate analysis to investigate their independent prognostic role. In particular, rCBV variation was used as a dichotomic parameter. All statistical analyses were performed using the R software (www.r-project.org).

## Results

### Clinical results

Patients' clinical and demographical baseline characteristics are reported in Table 1. In particular, MR showed in 31 cases (74%) local disease, in 6 (14%) multifocal and in 5 (12%) distant disease. No patient was previously treated with bevacizumab or other anti-angiogenic drugs.

**Table 1:** Patients characteristics at baseline.

Characteristic	No. of pts	%
Male	27	64
Female	15	36
Median age [all pts] (range)	53 (15–76)	
<40	9	21
40–60	25	59
>60	8	20
Median [all pts] (range)	70 (50–100)	
<70	5	12
70–80	33	78
90–100	4	10
De novo GBM	36	86
Secondary GBM	6	14
Disease recurrence: 1^st^/2^nd^	27/15	64/36
Radiotherapy	42	100
1^st^/2^nd^ line chemotherapy	42/15	100/36
Median tumor volume, cm^3^ (range)	22.29 (0.97–132.6)	
Local	31	74
Multifocal (Leptomeningeal dissem).	6	14
Distant	5	12
Diffuse	0	0

Abbreviations: cc, cubic centimetres; dissem, dissemination; MR, magnetic resonance; pts, patients.

During treatment three patients discontinued irinotecan before progression due to low tolerance and continued bevacizumab as monotherapy.

Tumor volumes at baseline were significantly lower in patients presenting local disease than in patients presenting distant or multifocal disease (median 17.2 versus 36.7 and 40.8 cm^3^, respectively; Mann Whitney *p* = 0.01).

Median follow-up was 33.5 weeks (range 9–111 weeks). At the time of this analysis, four of 42 patients were progression-free, three died before disease progression and four were alive.

Median OS was 35.0 weeks (CI 25.5–44.5); OS at 6 months was 66% (CI 52.0–80.0) and OS at 12 months 22% (CI 9.0–36.0). Median PFS was 20.0 weeks (CI 11.8–28.2), PFS at 6 months was 40%.

Median OS and median PFS were not significantly different when considering: sex, age ≥40 versus <40, age ≥60 versus <60, KPS ≥70 versus <70, and partial response versus stable/progressive disease according to RANO criteria [13].

**Figure 2:**
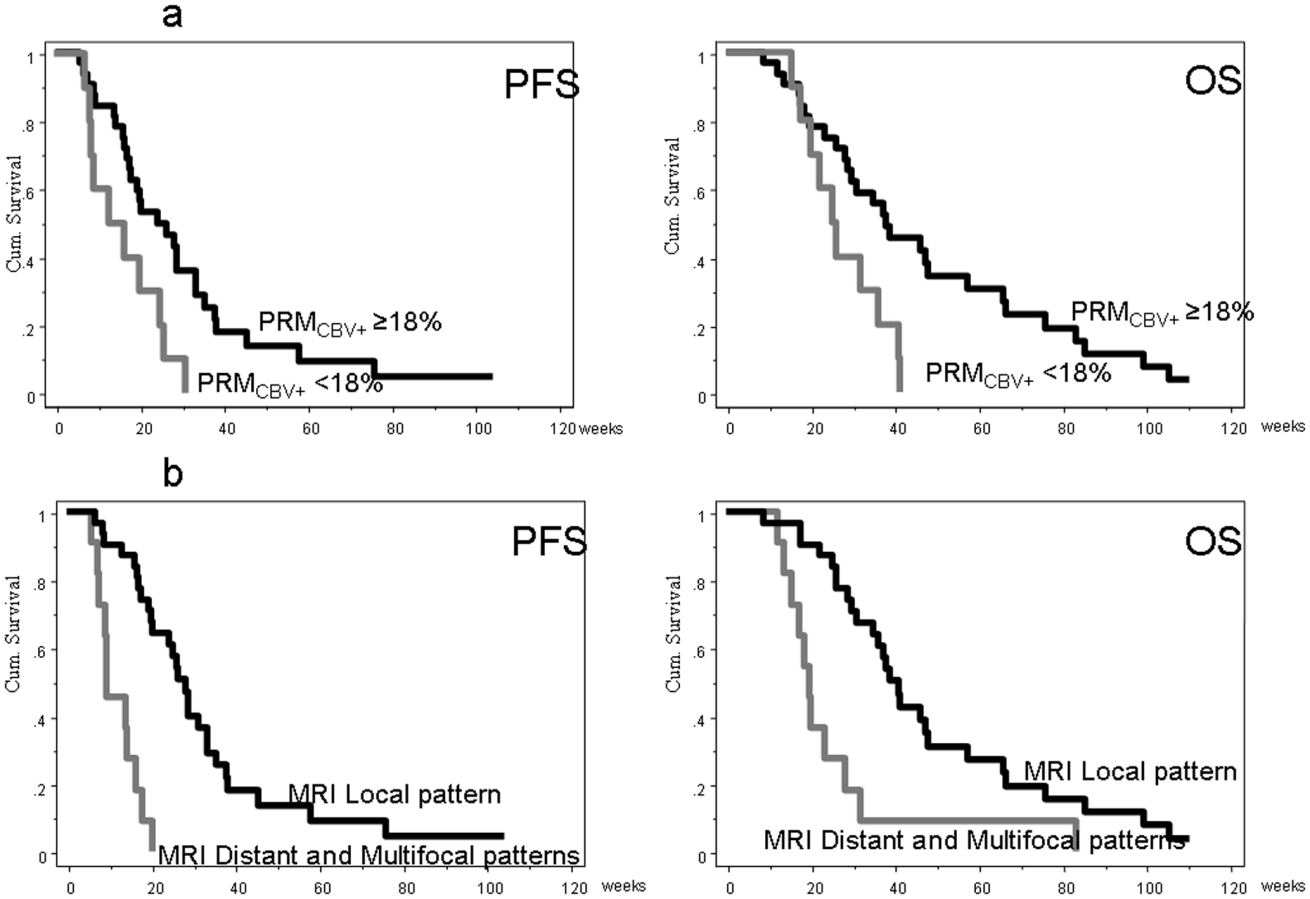
Correlations between PRM_CBV+_ higher than 18% and baseline magnetic resonance disease patterns and PFS/OS. A: Patients with PRM_CBV+_ higher than the first quartile, 18%, had longer survival than the others. B: Patients with local pattern of disease at baseline had longer PFS and OS than those with distant or multifocal disease.

Patients with local pattern of disease at baseline had longer PFS and OS than patients with distant or multifocal disease at baseline (PFS 28.0 versus 9.0 weeks, *p*<0.001; OS 41.0 versus 18.0 or 19.0 weeks respectively, *p* = 0.001. Figure 2).

All patients underwent MRI at baseline and 8 weeks after treatment onset; 32 patients were also assessed 16 weeks after treatment onset.

### Magnetic resonance results

MRI at 8 weeks after treatment onset showed partial responses in 9 cases, stable disease in 23 patients and progressive disease in 10. In this report we define patients showing progression at 8 weeks as *non-responsive*, and patients radiologically improved or with stable disease as *responsive*. No other partial or complete response was observed later.

The analysis of tumor responses to treatment at 8 weeks or later time points was available in 32 patients: other 10 patients died or interrupted treatment before progression or had incomplete neuroimaging.

Twenty patients (16 with local and 4 with multifocal disease) did not show changes when compared to baseline. Seven patients converted to a diffuse pattern of disease (6 patients starting from local and 1 from distant pattern), whereas 5 converted to multifocal disease (2 from local and 3 from distant disease).

### Analysis with the ROI method

Results are detailed in Table 2. Mean rCBV of all patients at baseline was 4.4 (±1.2 SD) (Table 2a).

**Table 2:** a) Mean rCBV max at different timepoints b) mean PRM_CBV_- and PRM_CBV_+ at different time points.

a.			
	No. of pts	Baseline rCBV	8 week-rCBV
*All patients*	42	4.4±1.2^a^	4.1±1.6 ^b^
Non-responsive	10	4.8±0.9	5.1±1.2
Responsive	32	4.2±1.3^c^	3.8±1.6^d^
*MR pattern at Baseline*			
Local	31	4.2±1.0^e^	4.0±1.5
Multifocal or Distant	11	4.9+1.6^f^	4.5±1.9
a–b *p* = 0.04; c–d *p* = 0.01; e–f *p* = 0.04.			

b.			
	No. of pts	PRM_CBV_-	PRM_CBV_+
*All patients*	42	18%±14%	30%±16%
Non-responsive	10	12%±11%	23%±19%
Responsive	32	19%±15%	33%±14%
*MR pattern at Baseline*			
Local	31	19%±15%	31%±16%
Multifocal or Distant	11	15%±10%	29%±18%

Patients with local disease at baseline showed significantly lower rCBV than patients with distant or multifocal disease at baseline (p = 0.041).

**Figure 3:**
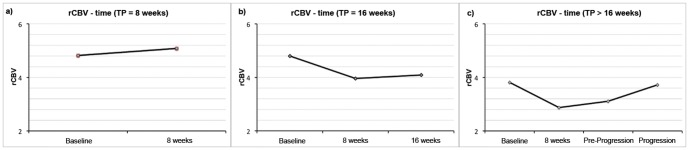
Mean rCBV changes according to treatment response within time. a) Mean rCBV remained elevated in non responsive patients who progressed after 8 weeks of treatment. b) Mean rCBV resulted in a significant decrease at 8 weeks, and in a new increase at 16 weeks, when progression occurred at 16 weeks. c) Mean rCBV showed a decrease at 8 weeks also in patients who progressed at 24 weeks or later but in the following MR performed before progression a light continuous increase of rCBV was observed.

In all patients mean rCBV decreased significantly at 8 weeks (4.4±1.2 SD versus 4.1±1.6 SD; p = 0.040), but was statistically unchanged at 16 weeks (3.7±1.3 SD; n.s.). The serial measurement of rCBV at all time points until progression, performed in 24 patients, showed that rCBV changes differently within time according to treatment response. After 8 weeks of therapy 32 responsive patients showed a significant decrease of rCBV with respect to the baseline (4.2±1.3 versus 3.8±1.6 p = 0.04), whereas 10 non responsive patients, who progressed at 8 weeks maintained elevated rCBV (4.8±0.9 versus 5.1±1.2, p = 0.38) (Figure 3a).

Patients who progressed after 8 weeks showed an initial significant decrease of rCBV, followed by a progressive tendency to increased rCBV as long as they approached tumour progression.

In particular, when progression occurred at 16 weeks (8 cases) a new increase in rCBV at 16 weeks occurred after a significant decrease at 8 weeks (Figure 3b); a decrease at 8 weeks in rCBV was also observed in patients who progressed at 24 weeks or later (6 cases), but in the following MR performed before progression a light continuous increase of rCBV was observed (Figure 3c).

The radiological pattern of disease at progression might influence rCBV changes. While patients with local, distant or multifocal disease at progression showed an initial rCBV decrease followed by a new increase, patients with diffuse disease at progression maintained a low rCBV (Table S1 in File S1).

### Parametric response maps (PRM)

Increased or decreased blood volume in PRM was defined as PRM_CBV+_ or PRM_CBV-,_ respectively, as in the work of Galbán et al [8]. At 8 weeks we observed a mean PRM_CBV+_ of 30%±16% and a PRM_CBV-_ of 18%±14% in all patients. Similar data were obtained at 16 weeks of treatment: PRM_CBV+_ was 32%±19% and PRM_CBV-_ was 17%±14% (Table 2b).

If we consider PRM results dividing patients into non-responsive and responsive, at 8 weeks we found a lower PRM_CBV+_ value in non-responsive than responsive (23%±19% versus 33%±14%, not significant) (Table 2b). Most of the non-responsive presented PRM_CBV+_ <18% (first quartile, *p* = 0.04).

We also examined perfusion at progression in 24 patients using PRMs: PRM_CBV+_ was 30%±20% and PRM_CBV-_ 20%±18%. No significant difference in PRM at progression was found dividing patients according to treatment response or pattern of disease at progression (see Table S2 in File S1).

### Correlation with survival

Median PFS and OS were longer in responsive patients (PFS: 8 versus 17 months, *p*<0.0001; OS:18 versus 39 months, *p*<0.0001).

Tumor volume higher than 75° percentile (44.5 cm^3^) was associated with significantly shorted PFS (14 versus 18 weeks, *p* = 0.023) and OS (24 versus 39.00 weeks, *p* = 0.049).

Using the classical ROI method rCBV values (at baseline and 8 weeks) did not correlate with PFS or OS. PRM analysis, on the contrary, did show correlations with survival. Patients with PRM_CBV+_ higher than 18% (first quartile) showed a significantly longer survival (Figure 2): median OS was 38.0 weeks in patients with PRM_CBV+_ ≥18% and 25.1 weeks in patients with PRM_CBV+_<18% (*p* = 0.016); median PFS was 24.3 weeks in patients with PRM_CBV+_ ≥18% and 13.1 weeks in patients with PRM_CBV+_ <18% (*p* = 0.045).

### Multivariate analysis

A multivariate analysis and a Cox proportional hazards regression analysis were performed on variables showing statistically significant differences at univariate analysis to investigate their independent prognostic role. Multifocal or distant pattern of disease at baseline and PRM_CBV+_<18% were independent predictors of shorter PFS (HR 8.2, *p* = <0.0001 and HR 3.2, *p* = 0.021, respectively).

Multifocal and distant pattern at baseline were the only independent predictors of OS (HR 3.1, *p* = 0.01), (Table 3).

**Table 3:** Univariate and multivariate analysis.

	Progression Free Survival		Overall Survival	
Model	Univariate *p* values	Multivariate *p* values	Univariate *p* values	Multivariate *p* values
Volume ≥44.47 cm^3^	0.02	n.s.	0.04	n.s.
Multifocal and Distant pattern	<0.0001	<0.0001	0.01	0.01
PRM_CBV+_<18%	0.045	0.02	0.016	n.s.

## Discussion

GBM is a tumor characterized by heterogeneous features with different regional expressions of potential therapeutic targets such as EGFR and VEGF [18], [19]. The pattern of microvascular proliferation can be various within the tumor with both simple, hyperplastic capillaries with increase endothelial cellularity and lumen patency, and complex, large collections of capillaries with partially thrombosed slit-like lumen, microvascular hyperplasia, resulting in minimal perfusion to the surrounding tumor tissue [20] [17].

MRI and Positron Emission Tomography (PET) can give detailed information about tumor heterogeneity. In particular, advanced MRI techniques could lead to a better microstructural and functional characterization of gliomas. Diffusion MRI giving information about the degree of cellularity in the different portions of tumoral and peritumoral areas could be predictive and prognostic in glioma and seems to correlate with survival in patients treated with bevacizumab [17], [21]–[23]. Spectroscopy MRI (H-MRS) can inform about metabolite concentration in the tumoral portions and could be an early indicator of response to antiangiogenic therapy [24], [25].

Dynamic Susceptibility Contrast-MRI (DSC-MRI) gives information about microvascular density and antiangiogenic therapy efficacy and could be helpful in tumor grading. In particular, rCBV may provide a prognostic information complementing histopathology [16], [26].

In our work we used DSC-MRI to evaluate the hemodynamic response over time in patients affected by recurrent GBM and treated with bevacizumab and irinotecan. We chose this technique because of its extended use in the clinical practice and due to the characteristics of rCBV. Indeed rCBV is a reliable indicator of microvascularization [27] and can be used to assess glioma grade [16], [28] and distinguish progression from pseudo-progression [29]. Moreover, some studies demonstrated that rCBV correlates with overall survival [27], [28], [30].

The most common methods to evaluate rCBV over time are the ROI-based and the histogram-based. The first one is highly user-dependent but allows a precise identification of the portion of the tumor to be analysed; on the other hand, it cannot accurately characterize the hemodynamic heterogeneity of high grade gliomas. The histogram-based method is less user-dependent and allows a better representation of the tissue heterogeneity, with similar sensitivity but higher specificity than the ROI method [8], [16]. Its main limitation is spatial localization: it gives information about glioma heterogeneity and might give indications about glioma grade, but it is not able to spatially localize regions where rCBV changes occur.

In this work the ROI method was used in comparison to PRMs. The PRMs [8], [9] is a voxel-wise technique estimating point by point the rCBV differences over time to better inquire the hemodynamic features of the tumor and to spatially localize the occurrence of hemodynamic changes. We compared PRMs with the classical ROI approach to investigate which one could better characterize the temporal variations of the tumor during therapy and have a better prognostic value.

The main result of the study is the correlation of PRMs with PFS at treatment onset. PRM_CBV+_ >18%, in particular, proved to be a valid prognostic marker of response whereas rCBV obtained by classical ROI showed no correlation with survival. These results are in accordance with those by Sorensen et al. [5], and Batchelor et al. [31] but different with respect to data published by Galbán et al. [8] where PRM_CBV-_ rather than PRM_CBV+_, was predictive of OS.

This discrepancy could be mostly due to the different type of therapy used in the two studies: radio-chemotherapy in the study of Galban et al [8], bevacizumab and irinotecan in our population. Ionizing radiation and classical chemotherapy tumor growth through the induction of DNA damnage, while Bevacizumab is a target therapy inducing inhibition of vessel expansion, regression of pre-existing vasculature and inhibition of bone marrow derived cell and/or endothelial progenitor.

Other relevant differences include different kinds of tumors (grade III and IV gliomas versus recurrent high grade gliomas) and time points considered: they studied subjects before therapy and 1–3 weeks after treatment onset, we studied our subjects before therapy and every 8 weeks until progression. More recently Galbán et al. showed the predictive value of the PRMs in association with ADC functional diffusion maps [32]. Using this method, they studied high grade gliomas treated with radio-chemotherapy before the beginning of therapy and three weeks after, finding that the combination of PRM_ADC+_ and PRM_CBV-_ has a predictive value, strongly correlating with OS at 1 year. Moreover, in this study patients received a treatment different from bevacizumab/irinotecan and were observed for a shorter duration.

Using PRMs Batchelor et al. [31] demonstrated that treatment with cediranib, a pan-VEGF receptor tyrosine kinase inhibitor, increases perfusion, in 50% of patients with newly diagnosed GBMs and that these patients survive 9-mo longer than those whose perfusion does not increase.

Differently from the PRMs method, the classical ROI approach did not provide a significant correlation with survival, in accordance with previous results by Galban et al. [8]. In addition, at 8 weeks the classical ROI method showed an initial decrease of the rCBV values, whereas the PRMs method showed greater PRM_CBV+_. The ROI method registered the mean rCBV variation in three ROIs placed in the points of maximum rCBV in the ehnancement region [16], whereas the PRMs consider a greater volume and analyze differences in a voxel-wise manner. Thus the first method is biased by the user ROI selection as it only takes into consideration the regions of maximum rCBV. Thus, mean rCBV after 8 weeks of treatment remained elevated in non responsive patients, but decreased in responsive patients, followed by a progressive tendency to increase as long as patients approached tumor progression.

On the contrary, the PRM method is less user-dependent as it considers the whole enhancement region, being unaffected by the selection criterias.

As previously reported [30], the PRMs tecnique is preferable because it offers the same sensibility but a higher specificity than the ROI classical approach. Interestingly, in non-responsive patients no major modification of perfusion was observed after treatment, suggesting that in non-responsive patients angiogenesis might be less VEGF dependent [33].

Even if the PRMs approach is affected by intrinsic limitations [8], [17], such as the need of a high quality image registration, it provided relevant information. Its potential to predict survival in recurrent GBM treated with bevacizumab and irinotecan, adds to previous results in high grade gliomas treated with radio-chemotherapy [8], making it a promising prognostic biomarker. Moreover, the tecnique (born with the analysis of ADC maps [17]) could be used in combination with other kind of sequences, such as the Dynamic Contrast Enhanced-MRI (DCE-MRI) to obtain more detailed informations about the biology of the disease under treatment.

## Supporting Information

File S1 **Supplementary tables.** Table S1, Mean rCBV changes according to radiological disease pattern at progression. Table S2, Mean PRM_CBV_- and PRM_CBV_+ changes during treatment.
(DOCX)
