# Supplementary material for: Parametric Response Maps of Perfusion MRI May Identify Recurrent Glioblastomas Responsive to Bevacizumab and Irinotecan
Source: PLoS One. 2014 Mar 27;9(3):e90535. doi: 10.1371/journal.pone.0090535 (PMC3968002; doi:10.1371/journal.pone.0090535)
Supplement: File S1 — Supplementary tables. Table S1, Mean rCBV changes according to radiological disease pattern at progression. Table S2, Mean PRMCBV- and PRMCBV+ changes during treatment. (DOCX) [file pone.0090535.s001.docx]

**Supplementary Table S1**

**Mean rCBV changes according to radiological disease pattern at progression**

| **Patients** |  | **Time point** | **Num Patients** | **rCBV mean** | ***P*** |
| --- | --- | --- | --- | --- | --- |
| All | Non-diffuse | Baseline | 17 | 4.42 (1.47) | 0.94 |
|  |  | Tprogression | 17 | 4.43 (1.23) |  |
|  | Diffuse | Baseline | 7 | 4.58 (1.19) | 0.37 |
|  |  | Tprogression | 7 | 4.06 (1.52) |  |
| Tp> 8 weeks* | Non -diffuse | Baseline | 8 | 4.23 (1.80) |  |
|  |  | 8w | 8 | 3.49 (1.26) | **0.01** |
|  |  | Tprogression | 8 | 4.12 (0.80) | 0.07 |
|  | Diffuse | Baseline | 6 | 4.57 (1.31) |  |
|  |  | 8w | 6 | 3.49 (1.30) | **0.03** |
|  |  | Tprogression | 6 | 3.68 (1.26) | 0.78 |

*(Tp 16 weeks+ Tp> 16 weeks)

**Supplemetary Table S2**

**Mean PRM_CBV_- and PRM_CBV_+ changes during treatment**

| Patients |  | Num Patients | Median (SD) | Wilcoxon P |
| --- | --- | --- | --- | --- |
| All | Tp-IBV | 27 | 40%(20%) |  |
|  | Tp-DBV | 27 | 15%(18%) |  |
| Tp=8 ws | 8w-IBV | 10 | 23% (19%) |  |
|  | 8w-DBV |  | 12% (11%) |  |
| Tp=16 ws | 8w-IBV | 8 | 36% (16%) |  |
|  | 16w-IBV |  | 43% (21%) | 0.263 |
|  | 8w-DBV |  | **20% (24%)** |  |
|  | 16w-DBV |  | **13% (17%)** | **0.017** |
| Tp = 24 weeks | 8w-IBV | 5 | 33% (7.7 %) |  |
|  | 16w-IBV |  | 30% (12 %) | 0.500 |
|  | 24w-IBV |  | 25% (21 %) | 1.00 |
|  | 8w-DBV |  | 18% (4.4 %) |  |
|  | 16w-DBV |  | 23% (14 %) | 0.22 |
|  | 24w-DBV |  | 40% (28 %) | 0.27 |
| Tp > 16 weeks | 8w-IBV | 6 | 34% (7.8 %) |  |
|  | 16w-IBV |  | 31% (11 %) | 0.463 |
|  | Progression-IBV |  | 42% (21 %) | 0.686 |
|  | 8w-DBV |  | 18% (6.1 %) |  |
|  | 16w-DBV |  | 25% (12 %) | 0.173 |
|  | Progression-DBV |  | 29% (25 %) | 0.345 |
